# Orally Administered Brain Protein Combined With Probiotics Increases Treg Differentiation to Reduce Secondary Inflammatory Damage Following Craniocerebral Trauma

**DOI:** 10.3389/fimmu.2022.928343

**Published:** 2022-07-06

**Authors:** Yang Cui, Lixia Xu, Fanchen Wang, Zhengang Wang, Xiaoguang Tong, Hua Yan

**Affiliations:** ^1^ Clinical College of Neurology, Neurosurgery and Neurorehabilitation, Tianjin Medical University, Tianjin, China; ^2^ Department of Neurosurgery, Hebei Yanda Hospital, Langfang, China; ^3^ Tianjin Key Laboratory of Cerebral Vascular and Neurodegenerative Diseases, Tianjin Neurosurgical Institute, Tianjin Huanhu Hospital, Tianjin, China; ^4^ Department of Neurosurgery, Affiliated Hospital of Weifang Medical University, Weifang, China; ^5^ Department of Neurosurgery, Tianjin Huanhu Hospital, Tianjin, China

**Keywords:** craniocerebral trauma, immune tolerance, regulatory T cell, secondary inflammation, orally

## Abstract

Craniocerebral trauma is caused by external forces that can have detrimental effects on the vasculature and adjacent nerve cells at the site. After the mechanical and structural primary injury, a complex series of secondary cascades of injury exacerbates brain damage and cognitive dysfunction following mechanical and structural primary injury. Disruption of the blood-brain barrier and exposure of brain proteins following craniocerebral trauma, recognition by the immune system triggering autoimmune attack, and excessive secondary inflammatory responses causing malignant brain swelling, cerebral edema, and subsequent brain cell apoptosis provide a new direction for the suppression of brain inflammatory responses in the treatment of craniocerebral trauma. We observed that CD4^+^T/CD8^+^T in peripheral blood T cells of craniocerebral trauma rats were significantly higher than those of normal rats, and the ratio of CD4^+^CD25^+^Foxp3 (Foxp3)^+^Regulatory T cell (Treg) was significantly lower than that of normal rats and caused increased secondary inflammation. We constructed a rat model of post-surgical brain injury and orally administered brain protein combined with probiotics, which was observed to significantly reduce CD4^+^T/CD8^+^T and induce T-cell differentiation into CD4^+^CD25^+^Foxp3^+^Treg, thus, reducing secondary inflammatory responses following craniocerebral trauma. However, collecting intestinal stool and small intestinal tissues for broad target metabolomics, 16s rRNA bacteriomics, and the combined analysis of intestinal tissue proteomics revealed that oral administration of brain protein combined with probiotics activates glycerophospholipid and vitamin B6 metabolic pathways to promote the production of CD4^+^CD25^+^Foxp3^+^Treg. Therefore, we propose the novel idea that oral administration of brain protein combined with probiotics can induce immune tolerance by increasing Treg differentiation, thus, reducing secondary inflammatory injury following craniocerebral trauma.

## Introduction

Traumatic brain injury (TBI), also called craniocerebral trauma, is characterized by a high disability and lethality rate and is a common and recurrent disease risking human health (1). The inflammatory response induced by TBI in the central nervous system (CNS) is mediated by both resident and peripheral immune cells (2, 3). Secondary damage is mediated by several pathways: (1) Excitotoxic theory triggered by excessive glutamate secretion (4–6); (2) free radical generation destroying neuronal proteins and their phospholipid membranes (7, 8); (3) local and systemic immune activation causing immune-inflammatory responses. Mechanical damage causes neuronal destruction and activation of microglia and astrocytes, releasing damage-associated molecular patterns (DAMPs) such as high migratory protein-1 (9–11), inflammatory vesicles, and brain-derived microparticles (BDMPs). Secondary injury causes leakage *via* disruption of the blood-brain barrier, allowing the movement of DAMPs, BDMPs, and other inflammatory molecules and cells into and out of the injured brain (12–14). These components initiate a series of responses in the brain and other remote organs and generate central and peripheral immune responses, which play a key role in the innate and adaptive immune responses during tissue repair (15, 16).

Regulatory T cells (Tregs) are a subpopulation of T cells representing 5-10% of circulating CD4^+^ T cells. They are important for maintaining immune homeostasis and suppressing excessive immune responses (17). Tregs are currently believed to mediate the immune system and prevent tissue damage secondary to excessive immune responses, thus, playing a key role in the pathogenesis of TBI (18–20). An important potential target for the current TBI therapy is the effective regulation of Treg functions. In previous studies, the sphingosine-1-phosphate receptor modulator fingolimod inhibited lymphocyte efflux from lymphoid tissue and reduced T-lymphocyte and natural killer (NK) cell infiltration, while increasing the numbers of Tregs and producing neuroprotective effects after TBI (21). In patients with stroke, the number of circulating Tregs decreases rapidly after stroke. This transient decline is followed by a significant and sustained increase in the Treg numbers for weeks (22). Animal studies have revealed that depletion of circulating Tregs severely increases brain damage and exacerbates the functional outcome of the middle cerebral artery following 7 days. In stroke, Treg reduces tPA-induced hemorrhage (23, 24).

There had been no effective treatments for craniocerebral trauma, thus, we were the first to introduce immune tolerance methods. We continuously explored various immune tolerance pathways for craniocerebral trauma, screened out the most suitable cost, promoted the pathway of the orally administered brain protein, and applied it in the clinic, which was recognized by the academic community (25–30). In response to the individual differences in efficacy in some patients, we recognized the key organ for generating immune tolerance, the intestine, which has a dysbiosis problem, and added probiotics to the process followed by orally administered brain protein and achieved considerably satisfactory results (31).

In this study, we explored the mechanism of orally administered brain protein combined with probiotics to promote T-cell homeostasis and immune tolerance in the treatment of craniocerebral trauma through multiple dimensions, such as efficacy, immune tolerance index assessment, broad target metabolomics sequencing, and T-cell differentiation and function. We explored the mechanism of orally administered brain protein combined with probiotics to promote T-cell homeostasis and immune tolerance for the treatment of craniocerebral trauma. We added the upgraded therapeutic mechanism of oral probiotics to our previously developed therapy of “oral autologous cerebrospinal fluid (containing a large number of brain proteins) causing immune tolerance to brain protein for the treatment of craniocerebral trauma” to clarify the principle of the interaction between brain protein, intestine, and immunity and provide theoretical and experimental support for additional probiotics.

## Materials and Methods

### Experimental Animals

Male adult Sprague Dawley (SD) rats were obtained from the Experimental Animal Center of the Chinese Academy of Military Medical Sciences. Animals were housed in a 12 h light-dark cycle at the Tianjin Huanhu Hospital Animal Center and kept in sterile ventilated/racked cages with free access to food and water. The experimental protocols were performed per the national legislation and relevant guidelines and approved by the Institutional Animal Care Committee of the Tianjin Key Laboratory of Cerebrovascular and Neurodegenerative Diseases.

### Surgical Brain Injury (SBI) Model

Male SD rats (SPF class; 220−250 g; Experimental Animal Center, Chinese Academy of Military Medical Sciences). A standardized SBI model was used according to a previous report. General anesthesia was induced by pre-surgical intraperitoneal injection of pentobarbital. The head and sternal stalk of the rats were excised. Further, each rat was fixed in a stereotaxic frame and the sterile skin over the skull was incised through a single sagittal incision along the biparietal suture line. A small piece of the skull (4 mm in diameter) was flattened and removed with a bone drill on the right side of the skull along 2 mm of the sagittal line and 1 mm of the coronal line. A dural dissection was performed, and 3×3 mm of brain tissue was removed by sharp dissection. Hemostasis was confirmed and the incision was closed following SBI. Brain protein and prebiotics were administered to the BP and PC groups, respectively, and brain protein combined with probiotics was orally administered to the TP group.

### Repeated Freeze-Thaw Method to Extract Brain Protein, and Administration of Brain Protein and Probiotics

The skin was exposed by injecting pentobarbital intraperitoneally following anesthesia. The rats were rapidly fixed on a stereotactic device and the neck was broken. Under aseptic conditions, the scalp was sagittally dissected, the skull was separated, and brain tissue was exposed and removed. Further, the surface of small blood vessels and meningeal tissue were carefully removed, brain tissue was cut under aseptic conditions, rapidly placed at -80°C for 30 min, reheated in a 37°C water bath for 5 min, and completely ground in a rod mill at 4°C. This procedure was repeated 3-5 times, and the absence of brain cell morphology was confirmed with an inverted phase-contrast microscope. Protein concentration was determined by UV spectrophotometry; the concentration of the solution was adjusted to 5 mg/mL using ultrapure water placed at -80°C. The BP group was gavaged with 4 mL brain protein, and the PC group was gavaged with 500 mg tablets of *Bifidobacterium lactis* combined daily; the rats in the combination group were gavaged with 4 mL brain protein and 500-mg tablets of *Bifidobacterium lactis* daily.

### HAPI Microglia Culture

HAPI microglia were purchased from Ze Ye Bio Inc and cell number ZY-C1276(China). HAPI cells were cultured normally in a complete medium containing Dulbecco’s modified Eagle medium (DMEM) (Gibco,United States), supplemented with 10% fetal bovine serum (FBS, Gibco, Invitrogen, CA, United States) and 100 U/mL penicillin/streptomycin(Gibco,United States), and maintained in a humidified environment at 37°C containing 5% CO_2_.

### IEC-6 Cells

Rat small intestinal epithelial crypt cells, IEC-6, (CRL 100548, Beijing Beina Biological Company, Beijing, China) were cultured in DMEM containing 10% fetal bovine serum (FBS, Gibco, Invitrogen, CA, United States), 2 mg/L insulin, 50 IU/mL penicillin, and 50 mg/mL streptomycin and incubated at 37°C with 5% CO_2_. The medium was changed after 48 h of initial cell placement.

### Preparation of Spleen T Cells

Following the execution, the rats were immersed in 75% alcohol for approximately 10 min; on a sterile ultra-clean table, the left side of the abdomen was cut open transversely with tissue scissors, and a dark red, long strip of spleen was observed behind the stomach, which was removed by sterile forceps and placed in phosphate-buffered saline (PBS) buffer. The spleen was placed between two layers of mesh and ground under appropriate pressure; the grinding solution was placed in a centrifuge tube, and 4 mL of GE Ficoll-Paque PLUS lymphocyte separation solution was added to resuspend the cells. The supernatant was discarded, and the cells were resuspended using RPMI 1640 containing 10% fetal bovine serum and inoculated in culture flasks at 37°C and 5% CO_2_ for 3 h. The suspended cells were subsequently transferred to new culture flasks to remove the adnexal growth of monocytes. Some of the suspended cells were collected for determination of the T-cell purity.

### Co-Culture of HAPI and Primary T Cells

HAPI cells were cultured in the absence or presence of interferon-gamma (IFN-γ,cat400-20-20UG,PeproTech,United States) (100 U/mL), granulocyte-macrophage colony-stimulating factor (GM-CSF,cat400-23-70UG,PeproTech,United States) (10 ng/mL), and Myelin basic phosphoprotein (MBP,ab167888) (0.1µg/mL) for 72 h. Further, IFN-γ and GM-CSF were removed by washing with PBS, and HAPI cells were co-cultured with T cells. Groups of rat spleen T cells were added to HAPI monolayers (IFN-γ and GM-CSF-treated HAPI cells) at a ratio of 1:2 and co-cultured for 72 h in a complete culture medium. Supernatants were collected for cytokine analysis.

### Co-Culture of IEC-6 and Primary T Cells

1-stearyl 2-oley l phosphatidylserine(PS (18:0/18:1),cas321883-23-2,Avanti,United States) was dissolved in DMSO and added to the complete medium, and the concentration was adjusted to 500 nM. Rat primary T cells were co-cultured with IEC-6 cells at a ratio of 1:2 in a complete medium for 72 h. T cells were collected for flow cytometric assay, and total IEC-6 protein was extracted and detected by western blot.

### Flow Cytometry

For each sample, 10^6^ cells were collected and washed with fluorescence-activated cell sorting (FACS) buffer (1% bovine serum albumin and 0.05% sodium azide in PBS). Cells were resuspended in 100 µL of FACS buffer containing 1 µg of antibody, incubated on ice for 30 min, and resuspended in 500 µL of FACS buffer for analysis within 1 h. Coulter FACS equipment and FlowJo analysis software were used.

### Enzyme-Linked Immunosorbent Assay (ELISA)

The concentrations of cytokines, tumor necrosis factor (TNF)-α, interleukin (IL)-1β, and IL-6, were measured by ELISA kits (Lianke Biologicals, Hangzhou, China). One hundred microliters of each standard and specimen were added to the microtitration plate first, and further, 50 µL of enzyme conjugate was added to the wells. The plate was incubated for 60 min at 37°C. The incubation solution was removed, and the microtiter wells were rinsed 5 times with 1× washing buffer. Further, 100 µL of color development reagent was added to each well and gently stirred for 5 s. Following incubation at 37°C for 15 min, 100 µL of termination solution was added to each well to stop the colorimetric reaction. The absorbance at 450 nm was measured immediately using a micro-plate reader (Biosciences Pharmingen, USA).

### Western Blotting

Cells were lysed using radioimmunoprecipitation assay buffer per the manufacturer’s protocol (SolarbioCo., Beijing, China). Protein (50 μg/lane) was added to 12% sodium dodecyl sulfate-polyacrylamide gel electrophoresis and further transferred to membranes (polyvinylidene fluoride, Merck Millipore). Protein blots on membranes blocked by bovine serum albumin were incubated with antibodies against Lpin1, Pld1 (1:500 dilution; Jiangsu Affinity Biotechnology Co., Ltd.), or β-microtubulin (1:500 dilution; CST, USA) overnight. Following incubation with horseradish peroxidase-linked goat anti-rabbit immunoglobulin G (CST, USA) for 60 min, the membranes were exposed to potentiating chemiluminescent reagents.

### Immunohistochemistry

Immunohistochemical assays were performed by the Department of Pathology, Circle Lake Hospital. Rabbit polyclonal anti-ZO-1, occludin antibody (Abcam,UK) was used. Positive sections were scored independently by two experienced pathologists for comprehensive scoring.Staining scoring criteria: two pathologists conducted double-blind radiographs, 10 fields were randomly collected under the microscope, and the percentage of positive cells under the microscope and staining intensity were scored.(1) If the proportion of pigmentation cells is less than 5%, 0 points, 5%-25%, 26%-50%, 3%-75%, and more than 75%, 4 points.(2) Undyed score is 0, light yellow score is 1, brown-yellow score is 2, tan score is 3.(3) The two scores are multiplied by the positive grade: 0 is negative (-), 1-4 is weak positive (+), 5-8 is positive (++), 9-12 is strong positive (+++).

### 16s rRNA Gene Analysis (http://www.ncbi.nlm.nih.gov/bioproject/834629)

We selected 50 samples for taxonomic analysis of the 16S amplicons, including 6 groups of mice cultured for 3 days in probiotic, 6 groups of SBI samples cultured for 3 days, 5 groups of brain protein combined with probiotics samples cultured for 3 days, 6 groups of brain protein samples cultured for 3 days, 6 groups of probiotic samples cultured for 7 days, 3 groups of brain protein samples cultured for 7 days, 5 groups of brain protein combined with probiotics samples cultured for 7 days, 6 groups of SBI samples cultured for 7 days, and 6 controls. The 16S rRNA gene sequencing protocol was adapted from the Earth Microbiome Project 1 and the Human Microbiome Project 2. For each sample, we generated approximately 10,000 read lengths. We further multiplexed and merged the read lengths using USEARCH version 7.03 and clustered sequences with 97% similarity into operational taxonomic units (OTUs) using the UPARSE algorithm. The OTU was further mapped to a subset of the SILVA database, which included sequences from the V4 region of the 16S rRNA gene to determine the classification. Abundance was subsequently recovered by demultiplexing read lengths mapped to UPARSE OTU to produce a final taxonomic profile. Additionally, 7-day samples with >1000 mapped read lengths were used for downstream analysis.

### Abundance Analysis of Differential Microbiome

We performed differential abundance analysis on the 16S rRNA data obtained above. Initially, we used the abundance normalization method, which converts the relative abundance of microorganisms using the square root of the inverse sine. Moreover, to visualize the abundance characteristics of different microorganisms, we used nuclear density plots to depict the sample profile. For each microorganism, we scaled the fraction above the detection limit (non-zero) using the Sheather-Jones method 6 for the density estimate of the microorganism, such that the maximum density of the plot spans the distance from the baseline for a given sample. Samples below the detection limit are represented as bars on the left, where the number of bars spanning the distance between sample baselines indicates all zeros. Density estimates are further scaled by the fraction of non-zero samples to accurately represent the relative density differences among groups with different zero fractions.

### Expression Analysis of Differential Metabolome and Proteome

We first used the ERgene7 library in Python to perform endo-reference normalization analysis for the metabolic and protein groups separately to eliminate the batch effect of the samples. Second, we calculated the expression fold change between the experimental and control groups using FC and used Wilcoxon9 in the SciPy8 library in Python to perform a non. The significance threshold was false discovery rate (FDR) *P*<0.05 and Log2FC >0.5 for the metabolites and FDR *P*<0.05 and Log2FC >0.25 for the proteins.

### Multi-Omics Analysis of Metabolome and Microbiome

To identify the microbial features associated with the experimental and control groups and the metabolites that may be produced by them, we measured the probability of co-occurrence between data from 16S microbiomics and metabolites obtained from metabolomics data. We used mmvec version 1.0.210 to construct conditional probability matrices for the log-transformation of metabolites into microorganisms from each histological feature. A microbial-metabolite network was constructed using conditional probabilities >0.5 as the threshold.

### Multi-Omics Analysis of Metabolome and Proteome

To identify pathways associated with the metabolome and proteome, we used Joint-Pathway Analysis 11 of MetaboAnalyst for joint enrichment analysis of the proteome and metabolome.

### Multi-Omics Similarity Analysis

To confirm the relationship between probiotics and brain protein combined with probiotics and brain protein and brain protein combined with probiotics for the microbiology, metabolism, and protein groups, we used Pearson coefficient S12 to measure the similarity between the two groups, analyzed confidence intervals and *P*-values, and used linear regression to calculate the correlation between the two groups for controlled validation.

### Statistical Analysis

Multiple comparisons among the experimental groups were performed using ANOVA followed by *post hoc* tests. Comparisons between two experimental groups were made using Student’s t-test. *P*<0.05 was considered statistically significant.

## Results

### Combined Orally Administered Brain Protein and Probiotics Can Decrease CD4^+^T/CD8^+^T in Peripheral Blood T Cells of SBI Rats

**Figure 1:**
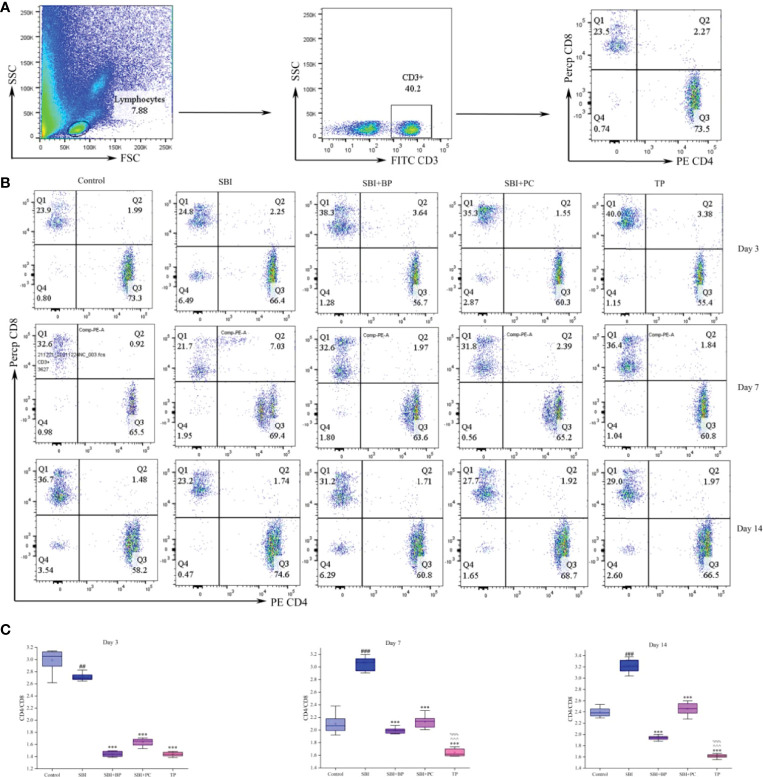
Oral brain protein combined with probiotics reduces CD4^+^T/CD8^+^T in peripheral blood of craniocerebral injured rats. **(A)** Representative gating strategy for the FACS analysis of CD3^+^CD4^+^CD8^+^ in rat blood cells. **(B)** Peripheral blood CD4^+^T/CD8^+^T was elevated in rats 3, 7, and 14 days after SBI and decreased after oral administration of ceruloplasmin, probiotics, and the combination (n = 6-8). **(C)** The CD4 relative intensities were compared with CD8 and represented by the mean ± SD. ^##^ *p* < 0.01, ^###^ *p* < 0.001 *vs.* Control, ***p < 0.001 *vs.* SBI, ^%%%^ *p* < 0.001 *vs.* SBI+BP, ^^^^^ *p* < 0.001 *vs.* SBI+PC (n = 3).

Forty experimental rats were randomly divided into five groups with eight rats in each, namely the control group (control), the post-surgical brain injury group (SBI), the post-surgical brain injury with orally administered brain protein group (SBI+BP), the post-surgical brain injury with orally administered probiotics group (SBI+PC), and the post-surgical brain injury with the combination of orally administered brain protein and probiotics group (TP). These rats were observed and executed in 3, 7, and 14 days. In the three-time gradients, CD4^+^T/CD8^+^T significantly increased in the SBI group, and following oral administration of brain protein and probiotics alone, the CD4^+^T/CD8^+^T significantly increased in the SBI group. CD4^+^T/CD8^+^T decreased following oral administration of brain protein and probiotics alone, and the most significant decrease in CD4^+^T/CD8^+^T was observed following combined oral administration of brain protein and probiotics (**Figure 1**).

### The Combination of Oral Brain Protein and Probiotics Increased CD4^+^CD25^+^Foxp3 (Foxp3)^+^Treg Expression in Peripheral Blood and Spleen

**Figure 2:**
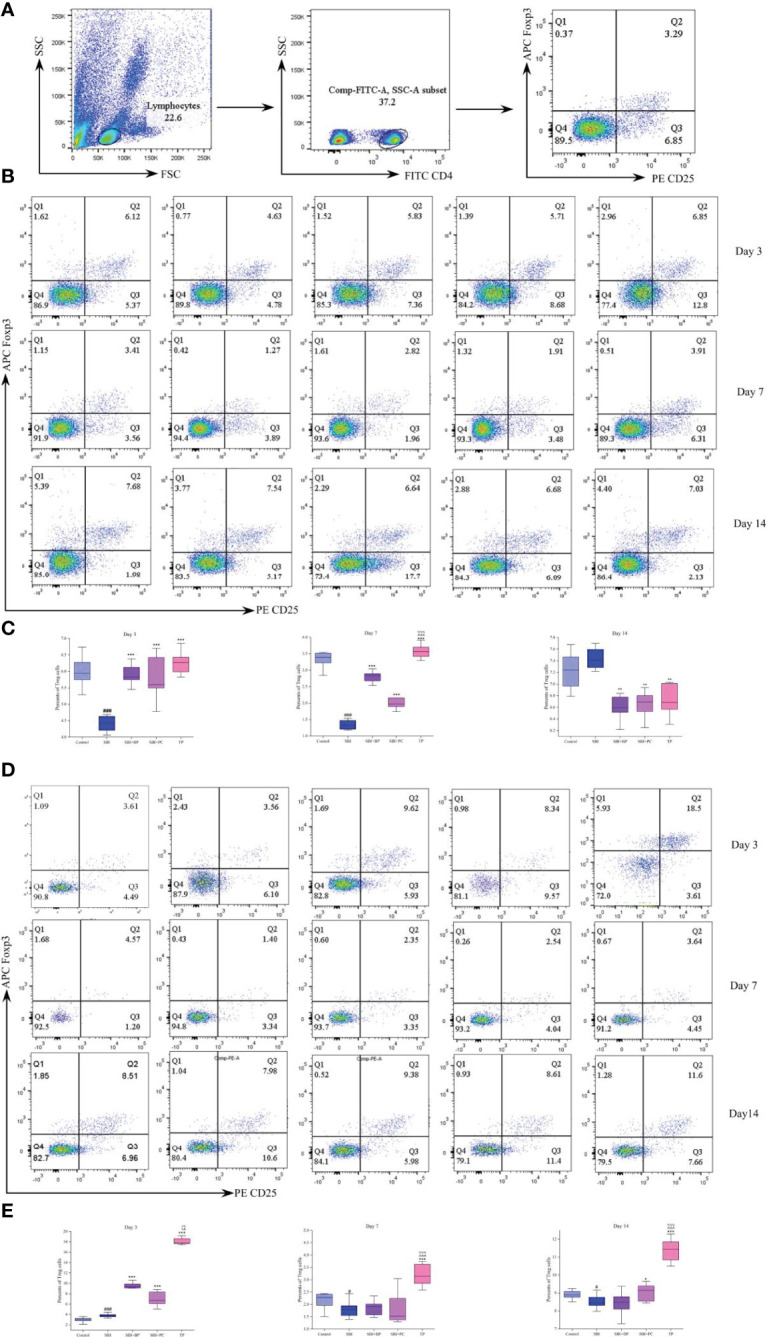
Oral brain protein combined with probiotics elevates CD4^+^CD25^+^Foxp3^+^Treg in peripheral blood and spleen of SBI rats. **(A)** Representative gating strategy for the FACS analysis of CD4^+^CD25^+^Foxp3^+^Treg. **(B)** Rat peripheral blood Treg percentage decreased after SBI and increased in the SBI+BP, SBI+PC and TP groups on days 3 and 7; Treg increased in the SBI group after 14 days. **(C)** Percents of Treg cells represented by the mean ± SD.^###^ *p* < 0.001 *vs.* Control, **p < 0.01, ***p < 0.001 *vs.* SBI, ^%%%^ *p* < 0.001 *vs.* SBI+BP, ^^^^^ *p* < 0.001 *vs.* SBI+PC (n = 3). **(D)** Rat spleen Treg percentage decreased after SBI and increased in SBI+BP, SBI+PC and TP groups. **(E)** Percents of Treg cells represented by the mean ± SD.^#^ *p* < 0.05 *vs.* Control, *p < 0.05, ***p < 0.001 *vs.* SBI, ^%%%^ *p* < 0.001 *vs.* SBI+BP, ^^^^ *p* < 0.01, ^^^^^ *p* < 0.001 *vs.* SBI+PC (n = 3).

Further, we isolated peripheral blood and spleen T cells from each group of rats and the proportion of CD4+CD25+Foxp3+Treg was tested by flow cytometry. The peripheral blood levels were elevated in the SBI+BP, SBI+PC, and TP groups compared with the SBI group on days 3 and 7, with the TP group being the most statistically significant among all three groups (**Figures 2A–C**). The percentage of Treg in the SBI group increased the most among all groups after day 14 (**Figures 2B, C**), and this result was associated with the subsidence of the edema period and the recovery of immunity following SBI. The results of Treg in the spleen revealed that Treg percentage in the TP group exhibited the highest expression in all three-time gradients whereas there was no statistically significant difference among SBI, BP, and PC groups as time lengthened (**Figures 2D, E**). The results of this part of the experiment revealed that combined oral brain protein and probiotics significantly increased the expression of CD4^+^CD25^+^Foxp3^+^Treg.

### CD4^+^CD25^+^Foxp3^+^Treg Reduces the Secondary Inflammatory Response Following Co-Culture With M1-Type HAPI

**Figure 3:**
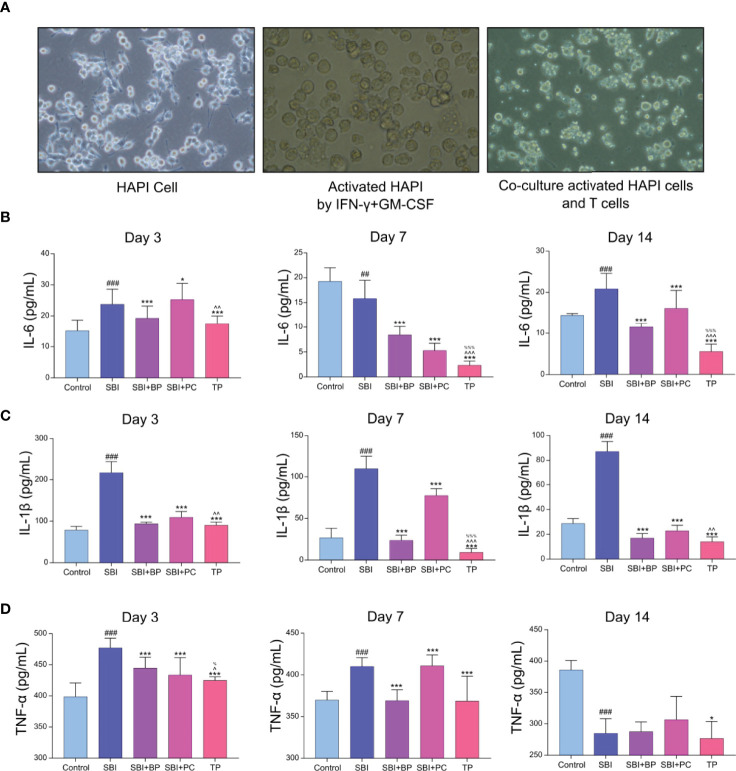
Treg decreased the inflammatory response after co-culture with activated HAPI. **(A)** The addition of IFN-γ+GM-CSF caused amoeboid changes in the cells after HAPI activation, and after co-culture with T cells, the T cells tended to the activated HAPI. **(B)** Pro-inflammatory factors of IL-6 was tested by ELISA kits. ^##^ *p* < 0.01, ^###^ *p* < 0.001 *vs.* Control, *p < 0.05, ***p < 0.001 *vs.* SBI, ^%%%^ *p* < 0.001 *vs.* SBI+BP, ^^^^ *p* < 0.01, ^^^^^ *p* < 0.001 *vs.* SBI+PC (n = 6). **(C)** Pro-inflammatory factors of IL-1β was tested by ELISA kits. ^###^ *p* < 0.001 *vs.* Control, ***p < 0.001 *vs.* SBI, ^%%%^ *p* < 0.001 *vs.* SBI+BP, ^^^^ *p* < 0.01, ^^^^^ *p* < 0.001 *vs.* SBI+PC (n = 6). **(D)** Pro-inflammatory factors of TNF-α was tested by ELISA kits. ^###^ *p* < 0.001 *vs.* Control, *p < 0.05,***p < 0.001 *vs.* SBI, ^%^ *p* < 0.05 *vs.* SBI+BP, ^^^ *p* < 0.05 *vs.* SBI+PC (n = 6).

To verify whether CD4^+^CD25^+^Foxp3^+^Treg can attenuate the inflammatory response, we performed *in vitro* cellular experiments. We first cultured rat microglia line, HAPI, and added IFN-γ (100 U/mL) and GM-CSF (10 ng/mL) to induce HAPI activation in the M1-type (**Figure 3A**), thus, establishing a microglia model following craniocerebral injury. Second, primary T cells from the spleen of rats in each group on days 3, 7, and 14 were extracted and co-cultured with M1-type HAPI at a ratio of 1:2. The cell supernatants were collected following 3 days, and the concentrations of inflammatory cytokines, IL-1β, IL-6, and TNF-α, were measured using ELISA. The results revealed that their concentrations were significantly higher in the SBI group (**Figures 3B−D**); those of IL-1β, IL-6, and TNF-α were significantly lower in the TP group (**Figures 3B−D**). It demonstrated that CD4^+^CD25^+^Foxp3^+^Treg reduces secondary inflammation following craniocerebral injury.

### Orally Administered Brain Protein Combined With Probiotics Promotes ZO-1 and Occludin Expression, Thereby, Facilitating Blood-Brain Barrier Repair Following Craniocerebral Injury

**Figure 4:**
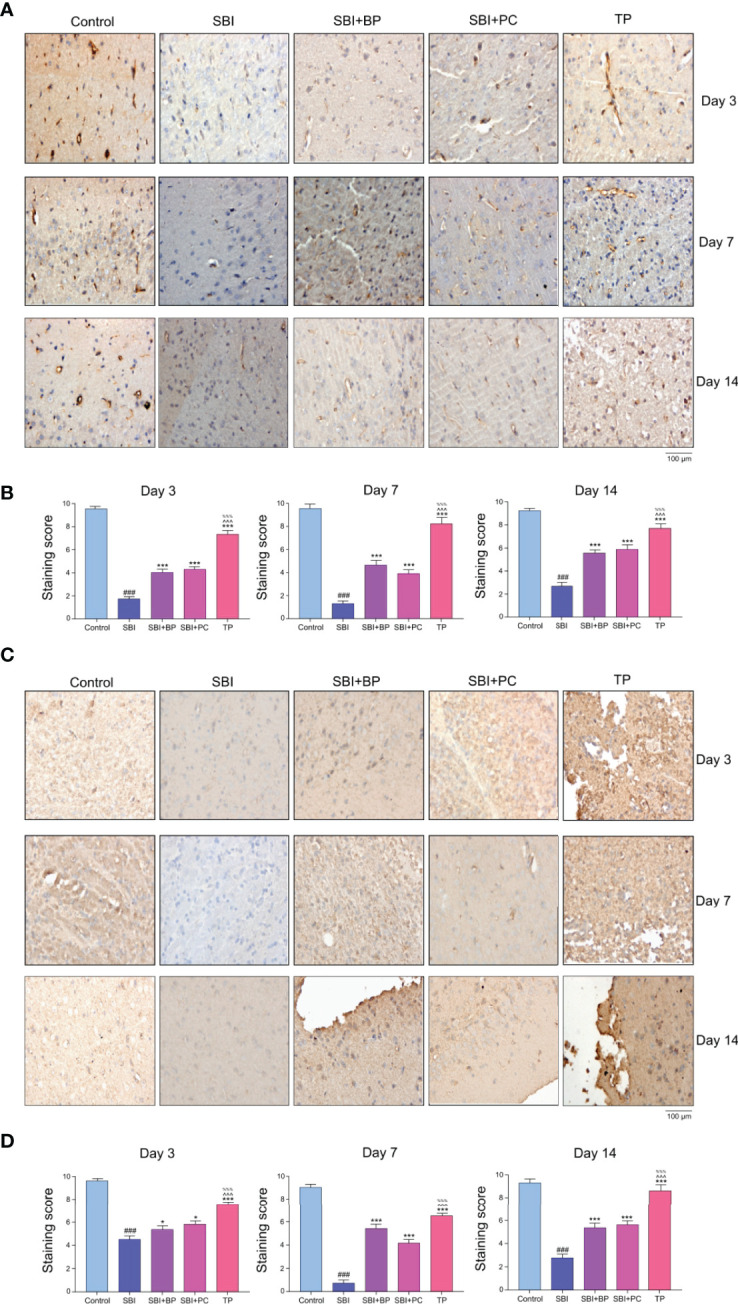
Oral brain protein combined with probiotics promotes ZO-1 and Occludin expression, thereby facilitating blood-brain barrier repair after craniocerebral injury. **(A)** Representative images of ZO-1 immunofluorescence staining in the brain tissues among the SBI, BP and combined groups at 3,7,14 days. **(B)** Immunohistochemical staining score. ^###^ *p* < 0.001 *vs.* Control, ***p < 0.001 *vs.* SBI, ^%%%^ *p* < 0.001 *vs.* SBI+BP, ^^^^^ *p* < 0.001 *vs.* SBI+PC (n = 6). **(C)** Representative images of Occludin immunofluorescence staining in the brain tissues among the SBI, BP and combined groups at 3,7,14 days. **(D)** Immunohistochemical staining score. ^###^ *p* < 0.001 *vs.* Control, ***p < 0.001 *vs.* SBI, ^%%%^ *p* < 0.001 *vs.* SBI+BP, ^^^^^ *p* < 0.001 *vs.* SBI+PC (n = 6), *p < 0.05 vs. SBI.

In a previous study, we demonstrated that astrocytes and microglia significantly were activated and secondary inflammatory damage aggravated following craniocerebral injury, and this phenomenon significantly reduced following oral administration of brain protein combined with probiotics (32). On the opening of the blood-brain barrier, peripheral T cells entered the injured area and reacted with activated microglia, thus, aggravating the secondary inflammation. To investigate whether the broken blood-brain barrier could be repaired following oral administration of brain protein and probiotics, we further verified the expression of tight junction proteins, ZO-1 and Occludin. The results revealed that after the establishment of the rat SBI model, the expression of brain tight junction protein, ZO-1 and Occludin, decreased in all three time gradients as the blood-brain barrier was broken whereas the expression of brain protein and probiotics alone increased to different degrees following oral administration. The increase was most significant after combined oral administration (**Figures 4A−D**). Thus, elevated Treg differentiation contributes to the repair of the blood-brain barrier. Further, to investigate how Treg differentiation is regulated following oral administration of ceruloplasmin and probiotics, we will predict the upstream mechanisms regulating it by combined analysis of intestinal flora, fecal metabolomics, and intestinal tissue proteomics.

### Global Landscape of Intestinal Flora, Fecal Metabolomics, and Proteomics

**Figure 5:**
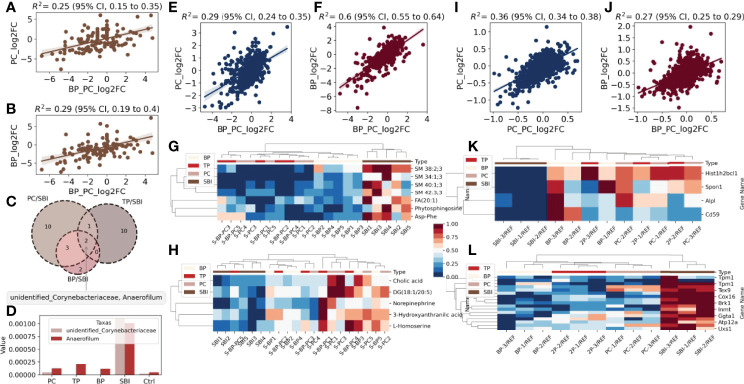
A global landscape of intestinal flora, fecal metabolomics and proteomics. Intestinal flora: **(A)** Similarity of PC and TP intestinal flora. **(B)** Similarity of intestinal flora between BP and TP. **(C)** venn diagram analysis of BP/SBI, TP/SBI, PC/SBI. **(D)** Comparison of the abundance of the common flora *Anaerofilum* with *Corynebacteriaceae* in the three samples.Metabolomics: **(E).** Metabolite similarity between PC and TP after co-administration. **(F)** Metabolite similarity between BP and TP after co-administration. **(G)** Heat map of abundance of metabolites with decreased co-expression in the three samples in each sample. **(H)** Heat map of the abundance of metabolites with increased co-expression in the three samples across samples. Proteomics: **(I).** Protein similarity between PC and TP after co-administration. **(J)** Protein similarity between BP and TP after co-administration. **(K)** Heat map of abundance of proteins with decreased co-expression in the three samples in each sample. **(L)** Heat map of the abundance of proteins with increased co-expression in the three samples in each sample.

**Figure 6:**
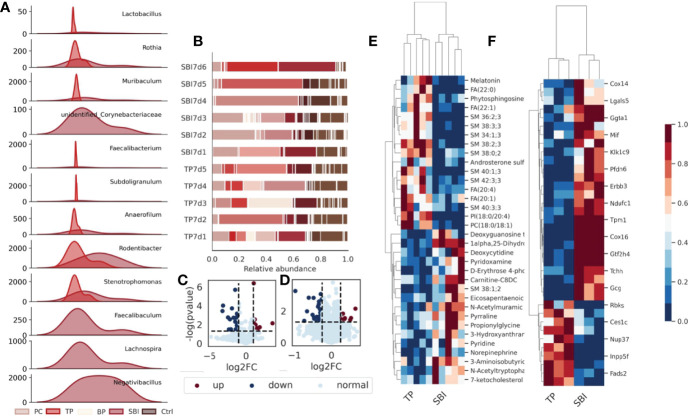
Intestinal flora, fecal metabolomics and proteomic profiles are affected during co-administration. **(A)** The abundance of a total of 9 flora changed significantly after the combined oral administration of BP and PC. **(B)** The composition of the flora changed after the combined oral administration. **(C)** Volcano plot showing significant changes in 157 proteins; **(D)** Volcano plot showing significant changes in 58 metabolites. **(E)** Heat map showing that 157 different proteins showed the same trend of change in each sample. **(F)** Heat map showing the same trend of 58 metabolites in each sample, blue represents low expression and red represents high expression.

To analyze the effect of oral administration of probiotic and brain protein alone versus co-administration of probiotic and brain protein, we initially performed an abundance analysis of the gut flora data. We observed that the similarity of intestinal flora was 0.25 (**Figure 5A**) between probiotic alone and co-administration of probiotic and brain protein and 0.29 (**Figure 5B**) between brain protein alone and co-administration of probiotic and brain protein. This indicates that the alteration of intestinal flora following co-administration is not consistent with the effect of single administration on the intestinal flora, and co-administration has a unique effect on intestinal flora. Additionally, we analyzed the altered intestinal flora for probiotic alone, brain protein alone, and co-administration of probiotic and brain protein and observed that *Anaerofilum* and Corynebacteriaceae exhibited significant changes in all three samples (**Figure 5C**). Their abundance revealed a decrease (**Figures 5D**, **6**), which was not significantly related to the administration of the drugs alone or in combination.

Subsequently, we analyzed the metabolome relationship between probiotic and brain protein administration alone versus co-administration of probiotic and brain protein. We observed that the similarity was 0.29 (**Figure 5E**) for metabolites of PC alone compared with co-administration of probiotic and brain protein and 0.6 (**Figure 5F**) for intestinal flora of brain protein alone compared with co-administration of probiotic and brain protein, suggesting that the metabolites affected by brain protein alone and co-administration of probiotic and brain protein are similar. Combined with the intestinal flora analysis, we concluded that this similarity was caused by metabolites secreted by two bacteria, *Anaerofilum* and *Corynebacteriaceae*. Additionally, we analyzed the metabolites that were co-regulated in abundance following probiotic and brain protein administration alone versus co-administration of probiotic and brain protein as SM; 38:2:3, SM; 34:1:3, SM; 40:1:3, SM; 42:3:3, FA; 20:1, phytosphingosine, and Asp-Phe (**Figure 5G**) and metabolites that were co-regulated in abundance DG (18:1/20:5) as 3-hydroxyanthranilic acid, norepinephrine, cholic acid, L-homoserine (**Figure 5H**), respectively.

We further analyzed the proteomic relationship between probiotics and brain protein administration alone and co-administration of brain protein and probiotics. We observed that the similarity of proteins was 0.36 for probiotics alone compared with co-administration of brain protein and probiotics and 0.27 for brain protein alone compared with co-administration of brain protein and probiotics, which indicates that the effect of administration alone and co-administration on proteins is not the same, and the proteins altered by co-administration have their uniqueness (**Figure 5I, J**). We analyzed the co-overexpression of Hist1h2bcl1, Alpl, Cd59, and Spon1 and that of Cox16, Tpm1, Tchh, Extl3, Brk1, Ggta1, Oma1, Atp12a, Uxs1, Tex9, Ndufc1, LOC691427, Snx15, Inmt, and Erbb3 (**Figures 5K, L**).

### Intestinal Flora, Fecal Metabolomics, and Proteomic Features Were Affected During the Co-Administration of Brain Protein and Probiotics

To further investigate the uniqueness of co-administration of brain protein and probiotics, we separately investigated the intestinal flora and fecal metabolomic and proteomic features of co-administration of brain protein and probiotics. We observed a total of 12 differential flora, including *Lactobacillus*, *Rothia*, *Muribaculum*, unidentified_Corynebacteriaceae, *Faecalibacterium*, *Subdoligranulum*, *Anaerofilum*, *Rodentibacter*, *Stenotrophomonas*, *Faecalibaculum*, *Lachnospira* and *Negativibacillus* in the intestinal flora(**Figure 6A**). The composition of the flora changed significantly following co-administration (**Figure 6B**). In metabolomics, a total of 58 metabolites revealed significant differences (**Figure 6C**), which exhibited the same trend of variation in each sample (**Figure 6E**). In proteomics, a total of 157 proteins revealed significant differences (**Figure 6D**) and similarly, exhibited the same variation in each sample (**Figure 6F**).Intestinal flora, fecal metabolomics, and proteomic features were affected during the co-administration of brain protein and probiotics.

### Following Co-Administration, Glycerophospholipid and Vitamin B6 Metabolic Pathways Were Altered and Interacted With Intestinal Flora in a Multi-Omics

**Figure 7:**
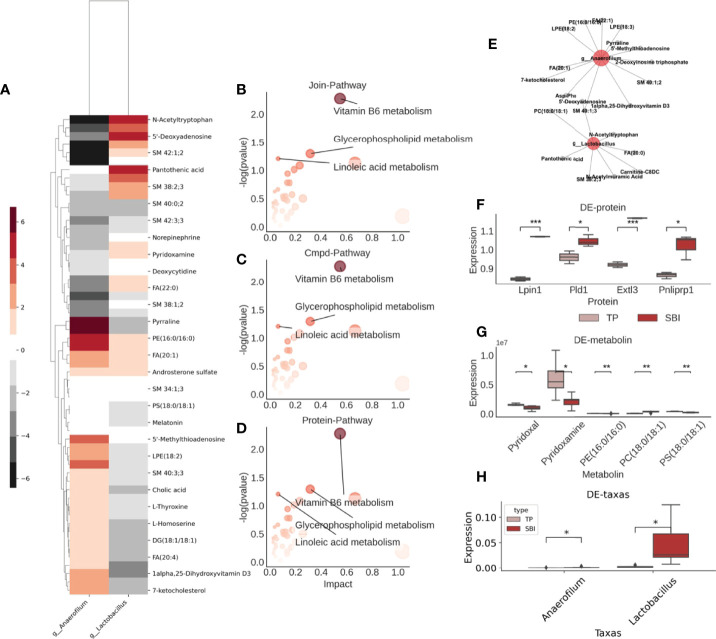
Glycerophospholipid metabolic pathways are altered after co-administration and interact with intestinal flora in multiple histologies. **(A)** Co-occurrence probabilities between intestinal flora and metabolites, where the higher the color the higher the co-occurrence probability and the lower the non-cooccurrence. **(B)** Results of the combined analysis of metabolite-related KEGG pathway and protein-related KEGG pathway, three pathways were found. **(C)** Visualization of metabolite-related KEGG pathway. **(D)** Visualization of protein-associated KEGG pathway. **(E)** Intestinal flora-metabolite interactions network. Red represents flora, gray represents metabolites. **(F)** Expression of differential proteins associated with the above pathway analysis. **(G)** Expression of differential metabolites associated with the above pathway analysis. **(H)** Comparison of the abundance of the four different bacterial groups. *p<0.05 ,**p<0.01,***p<0.005.

Subsequently, we explored the relationship between intestinal flora, metabolites, and proteins. Initially, we calculated the conditional probability of co-occurrence of intestinal flora and metabolites by the mmvec and selected the differential flora and metabolites among them, and we obtained a total of two flora of *Anaerofilum*, *Lactobacillus*, and their associated differential metabolites (**Figure 7A**). Besides, we combined 58 differential metabolites with 157 differential proteins using the MetaboAnalyst for metabolite-protein combination analysis and observed that glycerophospholipid and microbial B6 metabolic pathways were altered following co-administration of brain protein and probiotics (**Figures 7B–D**). Combining intestinal flora, fecal metabolomics, and proteomics, we obtained two pathways for co-administration to take effect (**Figure 7E**). One is the vitamin B6 metabolic pathway: A decrease in the abundance of *Anaerofilum* flora (**Figure 7H**) and a significant increase in the abundance of its co-occurring metabolite, pyridoxamine (**Figure 7G**), which in turn affects the vitamin B6 metabolic pathway; the other is the glycerophospholipid pathway, with a decrease in the abundance of *Anaerofilum* flora and a significant increase in the abundance of its co-occurring metabolite, PS (18:0/18:1) (**Figure 7G**). However, the expressions of related proteins Lpin1 and Pld1 decreased (**Figure 7F**), suggesting that PS (18:0/18:1) acted as an inhibitor to suppress the expression of related proteins.

### Vitamin B6 and PS (18:0/18:1) Promote T-Cell Differentiation to Treg *In Vitro*

**Figure 8:**
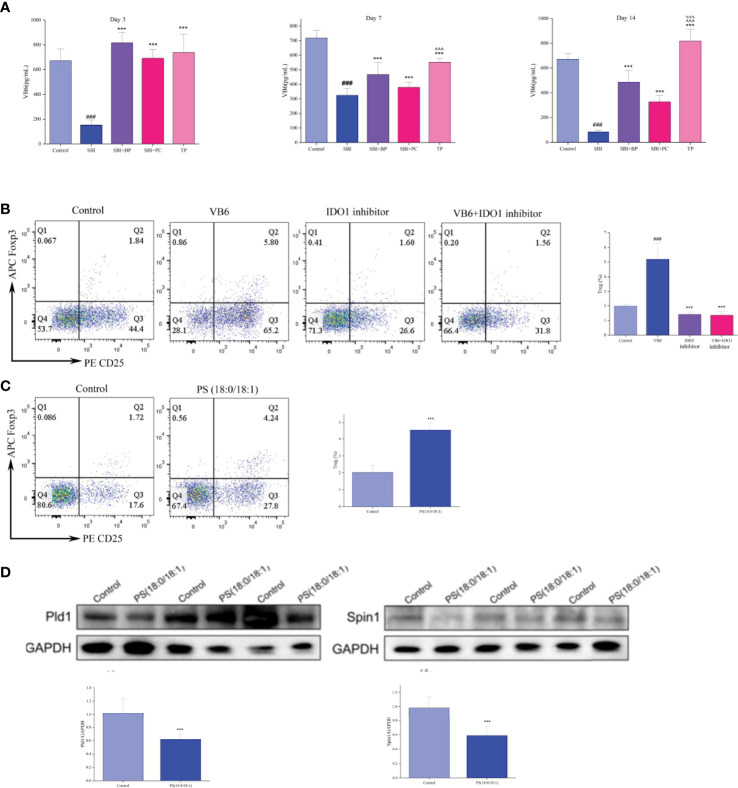
Vitamin B6 (VB6) and PS (18:0/18:1) promote T cell differentiation to Treg *in vitro*. **(A)** VB6 level was tested by ELISA kits. ^###^ *p* < 0.001 *vs.* Control, ***p < 0.001 *vs.* SBI, ^%%%^ *p* < 0.001 *vs.* SBI+BP, ^^^^^ *p* < 0.001 *vs.* SBI+PC (n = 6). **(B)** Percentage of Treg cells. ^###^ *p* < 0.001 *vs.* Control, ***p < 0.001 *vs.* VB6 (n = 6). **(C)** Percentage of Treg cells. ***p < 0.001 *vs.* Control (n = 6). **(D)** The Pld1 and Spin1 were tested by Western blot, and the relative intensity were represented by the mean ± SD.***p < 0.001 *vs.* Control, (n = 6).

Vitamin B6 intake and supplementation improve certain immune functions in vitamin B6-deficient humans and experimental animals. Inflammatory pathways dependent on vitamin B6 include vitamin B6 catabolism, a canine tryptophan metabolic pathway (33). Additionally, indoleamine-pyrrole 2,3-dioxygenase (IDO) 1, the key rate-limiting enzyme of tryptophan and kynurenine metabolic pathways, affects vitamin B6 metabolism and function (33); thus, we speculate that IDO1 may affect Treg differentiation by regulating vitamin B6 metabolism. We extracted normal rat spleen T cells for *in vitro* validation experiments, and T cells were inoculated in six-well plates according to 106 cells and divided into four groups: Control group, VB6 group (100 ng/mL), IDO inhibitor group (15 uM), and VB6 (100 ng/mL) + IDO inhibitor group (15 uM). Following 3 days of culture, flow cytometry was performed to detect the CD4^+^CD25^+^Foxp3^+^Treg percentage. Statistical analysis was performed after three independent experiments, which revealed that Treg percentage significantly increased in the VB6 group albeit decreased in the IDO1 inhibitor group and the VB6+IDO1 inhibitor group. This indicated that vitamin B6 promoted the differentiation of T cells into Treg whereas IDO1 inhibitor significantly inhibited this phenomenon (**Figure 8B**).

Subsequently, we measured the concentration of vitamin B6 in the plasma of rats in different time gradients using ELISA, and the concentration of vitamin B6 in rats in the SBI group decreased significantly whereas that in rats in the BP, PC, and TP groups increased significantly, with the highest in the TP group (**Figure 8A**). Based on these experimental results, we concluded that IDO1 can regulate the differentiation process of Treg through the vitamin B6 metabolic pathway.

In the validation experiments of the glycerophospholipid metabolic pathway, per the results of fecal metabolomics and intestinal tissue proteome (**Figures 7G, F**), we conjectured that PS (18:0/18:1) may increase Treg differentiation by inhibiting the expression of Lpin1 and Pld1. Initially, normal rat spleen T cells were extracted and co-cultured with rat intestinal epithelial cells, IEC-6, at a ratio of 1:2; a control group and PS (18:0/18:1) group (500 nM) were set up; T cells were collected following 3 days of Treg percentage assay; total protein of intestinal epithelial cells was extracted and protein expressions of Lpin1 and Pld1 were detected. Flow cytometry results revealed that the percentage of Treg cells in the PS (18:0/18:1) group was significantly higher than that in the control group (**Figure 8C**). Western blot results revealed that the expressions of Lpin1 and Pld1 were reduced in the PS (18:0/18:1) group (**Figure 8D**). This result validated the results of the combined multi-histochemical analysis and demonstrated that PS (18:0/18:1) increased Treg differentiation by decreasing the expression of Lpin1 and Pld1 in the glycerophospholipid metabolic pathway.

## Discussion

Brain protein exposure following craniocerebral trauma and recognition by the immune system triggers an autoimmune attack, and excessive secondary inflammatory response causes malignant brain swelling, cerebral edema, and subsequent brain cell apoptosis, providing a new direction for the suppression of brain inflammatory response in the treatment of craniocerebral trauma (34–36). Systemic immunosuppression using immunosuppression, radiation, and hypothermia, however, efficacious, has the risk of making the body immunocompromised and prone to inducing infection and distant tumorigenesis, which has become a bottleneck in its clinical translation and is applied by scholars in the treatment of spontaneous encephalomyelitis and organ transplantation to aid establishing immune tolerance (37, 38). Current clinical studies related to the immunosuppressive treatment of the nervous system include high-dose hormonal shock for craniocerebral trauma, the immunosuppressant, FK506, for spinal cord trauma, fingolimod therapy, and a novel drug restricting circulatory immune cells to the spleen following a cerebral hemorrhage. The efficacy of these immunosuppressive drugs utilized for the treatment of secondary inflammatory injury of the nervous system has been demonstrated, albeit immunosuppressants are expensive (e.g., FK506, fingolimod), with narrow safety profile (e.g., FK506), and with reduced overall immunity of the patient inviting infection (cut/wound, lung, urinary tract) (39). Thus, how to establish immune tolerance to brain proteins only, without affecting the body’s immunity to other antigens, is becoming an interesting research topic.

In animal models of craniocerebral trauma, we demonstrated that probiotic enrichment can contribute to the efficacy of immune tolerance to orally administered brain protein, which significantly improves intestinal barrier function, and that changes in the IDO-Kyn-AhR tryptophan pathway were initially identified (40). Although we observed that the tryptophan pathway was involved in the induction of immune tolerance by oral brain protein + probiotics, how combined oral administration modulates the IDO tryptophan pathway through the induced alterations in the intestinal flora-metabolite axis and how the intestinal flora-mediated IDO tryptophan pathway further modulates T-cell differentiation and function to induce immune tolerance was unclear. Although we performed basic and clinical studies on oral administration of autologous brain protein, thymic injection of autologous brain protein, hepatic portal vein injection of autologous brain protein, and peripheral intravenous injection of nanomaterial-coated autologous brain protein to establish immune tolerance for the treatment of craniocerebral trauma using various immune tolerance pathways and obtained recognition, the results lack potent indicators directly related to immune tolerance. Elevated peripheral blood CD4^+^T/CD8^+^T ratios in patients with craniocerebral trauma, indicating an active immune-inflammatory response (41, 42), and that oral brain protein + probiotics improved this phenomenon demonstrated by previous studies were verified in this study in a rat SBI model. This suggests that we need to further explore the effect of combined oral administration on T-cell differentiation and function and explore the process of this mechanism.

Treg is currently believed to play a key role in the pathogenesis of TBI by mediating the extent of immunity and preventing tissue damage secondary to excessive immune responses (16). How to effectively regulate the function and role of regulatory T cells would be an important potential target for current TBI therapy. In previous studies, fingolimod, a sphingosine-1-phosphate receptor modulator, inhibited lymphocyte efflux from lymphoid tissues and reduced T-lymphocyte and NK cell infiltration, while increasing Treg numbers and producing neuroprotective effects following TBI (21). Therefore, to verify whether brain protein combined with probiotics in the presence of intestinal microorganisms can increase peripheral CD4^+^CD25^+^Foxp3^+^Treg, we examined CD4^+^CD25^+^Foxp3^+^Treg in peripheral blood and spleen of rats in each group on days 3, 7, and 14 and observed that compared with the control group, Treg decreased significantly in the SBI group, SBI+BP group, SBI+PC group, and TP group. Treg was significantly higher, indicating that ceruloplasmin and probiotics regulate intestinal function and induce immune anti-inflammatory effect, that the effect was enhanced by the combination of the two, and the generation of immune tolerance.

CNS, especially the brain, is considered an immune organ, isolated from the immune system by the blood-brain barrier (43). Disruption of the blood-brain barrier by TBI exposes many brain proteins to the immune system, which activates immune cells, and induces cytokine and other inflammatory mediators to be produced and reach the site of injury, and excessive immune damage exacerbates the secondary inflammatory response, causing irreversible neurological deficits (44, 45). Studies have reported that controlling immune damage and secondary inflammatory responses significantly reduces brain edema and cerebral ischemia, impaired energy metabolism, calcium overload, and inflammatory factor stimulation (46–48). In previous studies, we demonstrated that oral brain protein inhibits glial cell activation and promotes brain tissue repair. This study revealed that oral brain protein combined with probiotics induces an increase in Treg differentiation and effectively repairs blood-brain barrier damage caused by trauma.

It is well-known that gut microbes have effective biological functions to influence the multi-systemic functions of the body. Thus, in which way do ceruloplasmin and probiotics achieve the induction of immune tolerance in the gut? In this study, a combined analysis of broad-target metabolomics, 16s rRNA bacteriomics, and intestinal tissue proteomics was performed to seek the upstream regulatory mechanisms that induce Treg differentiation. Integrative histology analysis revealed that the differential metabolites and intestinal epithelial proteins in the SBI and TP groups were mainly enriched in the vitamin B6 and glycerophospholipid metabolic pathways, and of five differential metabolites, two were mainly involved in vitamin B6 metabolism and three in glycerophospholipid metabolism. Two intestinal epithelial differential proteins were involved in glycerophospholipid metabolism. We further performed *in vitro* validation experiments based on the integromics results, presenting that oral ceruloplasmin combined with probiotics can inhibit the expression of Spin1 and pld1 and promote Treg differentiation through elevated PS (18:0/18:1) in the IDO1/vitamin B6 and glycerophospholipid metabolic pathways.Spin1 is overexpressed in many cancers and can promote cell proliferation, transformation, metastasis and chemical or radiation resistance (49). It is noteworthy that SPIN1 can regulate abnormal lipid metabolism by increasing triglyceride, cholesterol and lipid droplets in liver cancer cells, thus significantly promoting the proliferation of liver cancer cells (50), but there are few studies on THE differentiation of Treg by spin1 regulating lipid metabolism.Pld1 plays an important role in a variety of inflammatory and autoimmune diseases. In addition, ablation and inhibition of PLD1 inhibited the production of type II collagenous specific IgG2a autoantibodies and pro-inflammatory cytokines, while increasing the number of Treg cells and decreasing the number of Th17 cells in CIA mice (51), which was consistent with our results.

The active form of vitamin B6, pyridoxal 5′-phosphate (PLP), acts as a cofactor in over 150 enzymatic reactions. Plasma PLP levels have consistently been reported to be low under inflammatory conditions; there is a concurrent decrease in hepatic PLP levels, albeit little change in erythrocyte and muscle PLP levels and functional biomarkers of vitamin B6 (52, 53). Plasma PLP levels also predict the risk of chronic diseases (e.g., cardiovascular disease and certain cancers) and are negatively correlated with numerous inflammatory markers in clinical and population-based studies (54). Vitamin B6 intake and supplementation improve certain immune functions in vitamin B6-deficient humans and experimental animals (55). Zhang et al. reported that dietary interventions (including vitamins B6 and B12 and folic acid) reduced blood homocysteine levels albeit reduced inflammatory responses and corrected Treg/Th17 immune imbalances, thereby, improving brain tissue damage (56).

Glycerophospholipid metabolism, which regulates T cells (Treg), is more susceptible to lipid oxidation. Thus, high levels of lipids can reduce the number of effector T cells while promoting Treg production, and inhibition of the mitochondrial lipid transporter protein, CPT1A, can inhibit fatty acid oxidation and selectively block Treg differentiation (57). Increasing evidence suggests that the differentiation, polarization, distribution, signal transduction, and biological activity of immune cells can be regulated by lipid metabolism and bioactive lipids. The results of intestinal metabolomics combined with intestinal tissue proteomics analysis demonstrated that orally administered brain protein combined with probiotics significantly activated the glycerophospholipid metabolic pathway. We further selected lipid metabolites with large differences in ploidy PS (18:0/18:1) and lipid-like proteins, Spin1 and Pld1, for validation results of upstream and downstream pathway analyses. Pld1 inhibitors reportedly promote Treg differentiation *in vitro* to improve the validation response in autoimmune diseases (51); the effect of Spin1 on the proliferation and migration of tumor cells has been demonstrated in most tumor studies whereas studies on immune function have been rarely reported. We co-cultured normal rat spleen T cells with intestinal epithelial cells, IEC-6, and observed that the addition of PS (18:0/18:1) significantly promoted the differentiation of T cells into CD4^+^CD25^+^Foxp3^+^Treg. The expressions of Spin1 and Pld1 in intestinal epithelial cells decreased, which validated the integromic results and demonstrated that the glycerophospholipid metabolic pathway can effectively induce immune tolerance.

**Figure 9:**
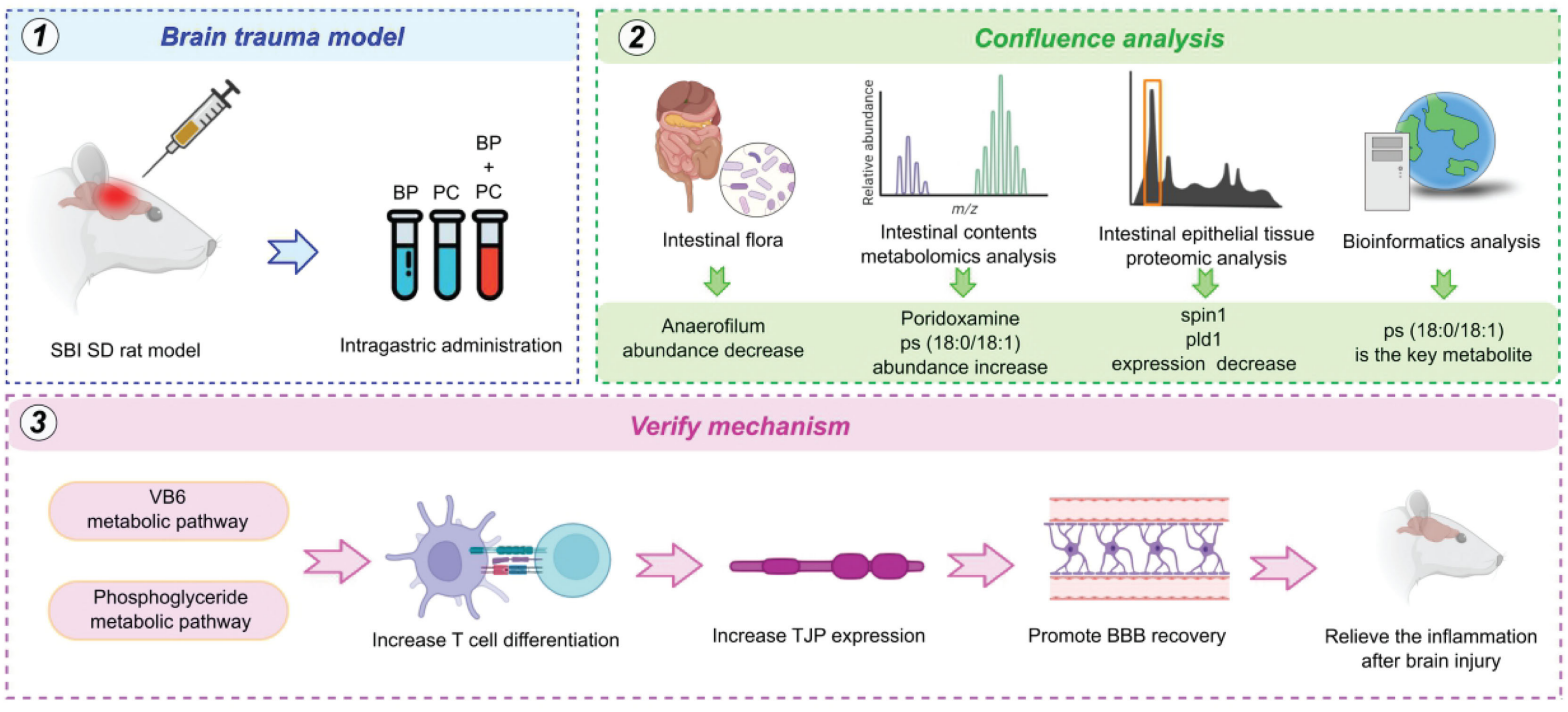
Workflow based on built brain trauma model, intestinal flora analysis, metabolomics, proteomics and bioinformatics analysis. The core mechanism of VB6 treatment were focus on VB6 metabolic pathway, phosphoglyceride metabolic pathway, including increase T cell differentiation, increase TJP expression, promote BBB recovery and relieve the inflammation after brain injury.

Conclusively, our study confirmed that oral ceruloplasmin combined with probiotics can promote the differentiation of peripheral T cells into Treg by altering intestinal metabolism, thus, inducing immune tolerance, repairing the blood-brain barrier, and reducing the secondary inflammatory response following craniocerebral trauma. These results are consistent with the findings of X et al. As an important immune organ of the body, the function of the intestine is regulated by the intestinal flora and metabolites. When brain protein enters the intestine, under the action of probiotics, a considerable number of pyridoxal molecules and various lipid molecules involved in the metabolic pathways of vitamin B6 and glycerophospholipids are produced, respectively, which induce an increase in CD4^+^CD25^+^Foxp3^+^Treg. This induces immune tolerance, reduces secondary inflammation following craniocerebral trauma through the regulation of the intestine-brain axis, promotes recovery and prognosis, and proves the safety and efficacy of orally induced immune tolerance, which has important clinical value and significance (**Figure 9**).

## Data Availability Statement

The datasets presented in this study can be found in online repositories. The names of the repository/repositories and accession number(s) can be found below: https://www.ncbi.nlm.nih.gov/bioproject/PRJNA832548, https://www.ncbi.nlm.nih.gov/bioproject/PRJNA834629.

## Ethics Statement

The animal study was reviewed and approved by Institutional Animal Care Committee of the Tianjin Key Laboratory of Cerebrovascular and Neurodegenerative Diseases.

## Author Contributions 

HY and XT designed the study. YC and ZW accomplished the SBI model, brain, feces, small bowel tissue, spleen collection, and western blot assy. YC and LX carried out peripheral blood and spleen lymphocytes collection, flow cytometry and immunohistochemistry staining. YC was responsible for cell culture, enzyme-linked immunosorbent assay, and bioinformatics data analysis. YC and FW performed data analysis. YC, LX, and FW wrote the manuscript. YC, LX, and FW contributed equally to this study. All authors read the final version and approved the submission and publication of the manuscript.

## Funding

Key Program of the Natural Science Foundation of Tianjin (grant numbers 20JCZDJC00730); Tianjin Health Science and Technology Project (Key Discipline Special Project).

## Conflict of Interest

The authors declare that the research was conducted in the absence of any commercial or financial relationships that could be construed as a potential conflict of interest.

## Publisher’s Note

All claims expressed in this article are solely those of the authors and do not necessarily represent those of their affiliated organizations, or those of the publisher, the editors and the reviewers. Any product that may be evaluated in this article, or claim that may be made by its manufacturer, is not guaranteed or endorsed by the publisher.

## Supplementary Material

Supplementary Experiment Oral brain protein combined with probiotics elevates CD4^+^CD25^+^Foxp3^+^Treg in brain of SBI rats. **(A)** Rat brain Treg percentage decreased after SBI and increased in the SBI+BP, SBI+PC and TP groups on days 7; **(B)** Percents of Treg cells represented by the mean ± SD. ****P* < 0.0001, *****P* < 0.00001.
