# Supplementary material for: Orally Administered Brain Protein Combined With Probiotics Increases Treg Differentiation to Reduce Secondary Inflammatory Damage Following Craniocerebral Trauma
Source: Front Immunol. 2022 Jul 6;13:928343. doi: 10.3389/fimmu.2022.928343 (PMC9298786; doi:10.3389/fimmu.2022.928343)
Supplement: Supplementary Experiment — Oral brain protein combined with probiotics elevates CD4+CD25+Foxp3+Treg in brain of SBI rats. (A) Rat brain Treg percentage decreased after SBI and increased in the SBI+BP, SBI+PC and TP groups on days 7; (B) Percents of Treg cells represented by the mean ± SD. ***P < 0.0001, ****P < 0.00001. [file Presentation_1.pdf]

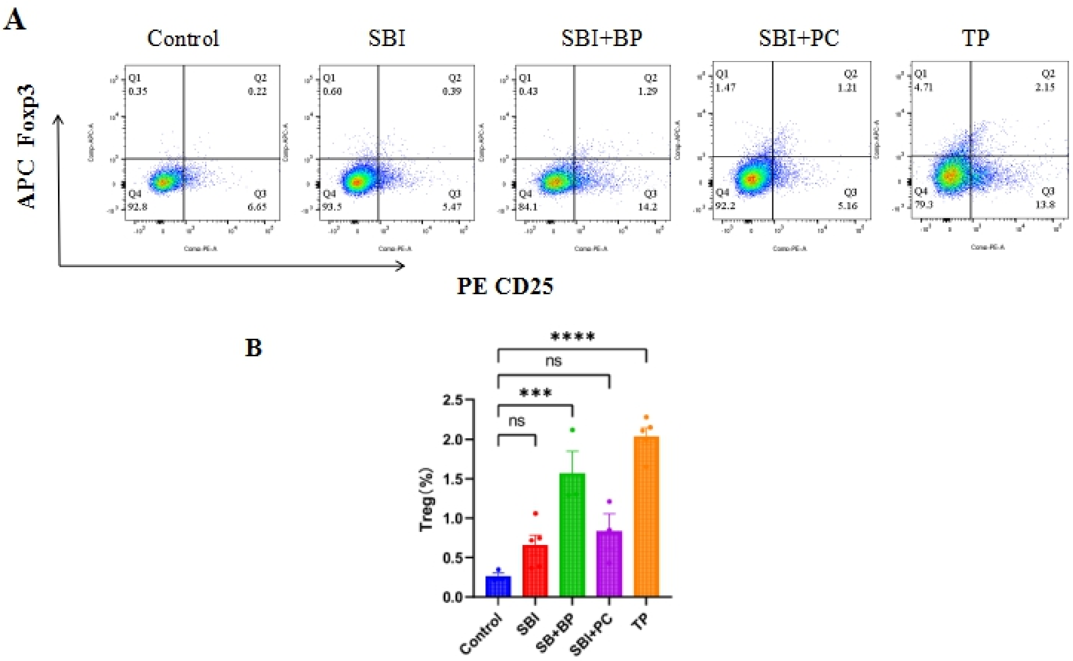

Oral brain protein combined with probiotics elevates $CD4^+CD25^+Foxp3^+$Treg in brain of SBI rats.(A) Rat brain Treg percentage decreased after SBI and increased in the SBI+BP, SBI+PC and TP groups on days 7;**(B)**Percents of Treg cells represented by the mean ± SD.***P＜0.0001，****P＜0.00001.
