# Supplementary figures and images for: Orally Administered Brain Protein Combined With Probiotics Increases Treg Differentiation to Reduce Secondary Inflammatory Damage Following Craniocerebral Trauma
Source: Front Immunol. 2022 Jul 6;13:928343. doi: 10.3389/fimmu.2022.928343 (PMC9298786; doi:10.3389/fimmu.2022.928343)

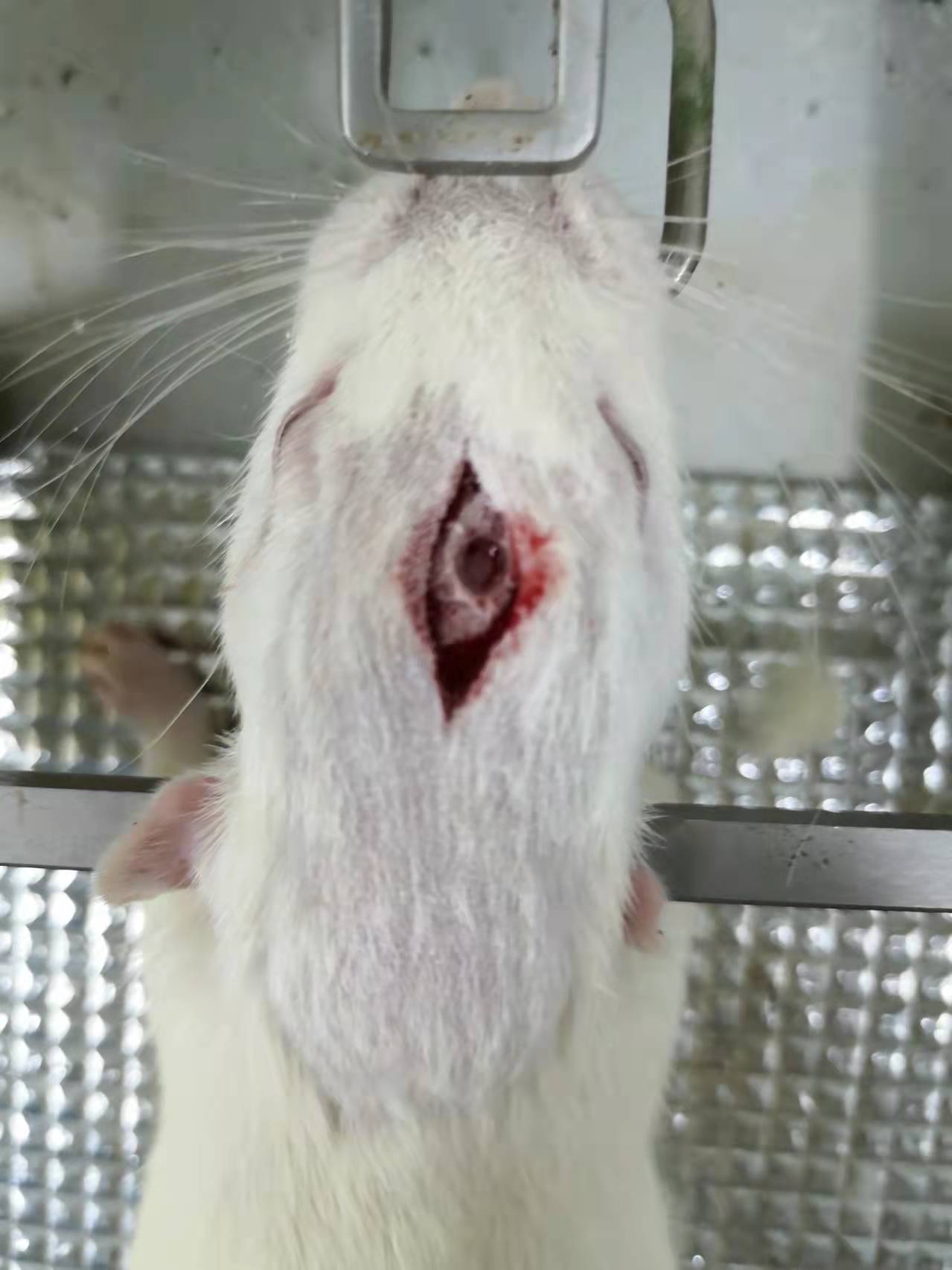

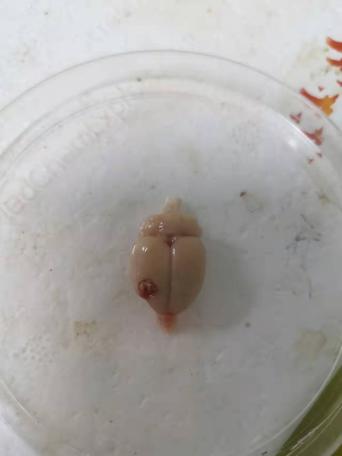

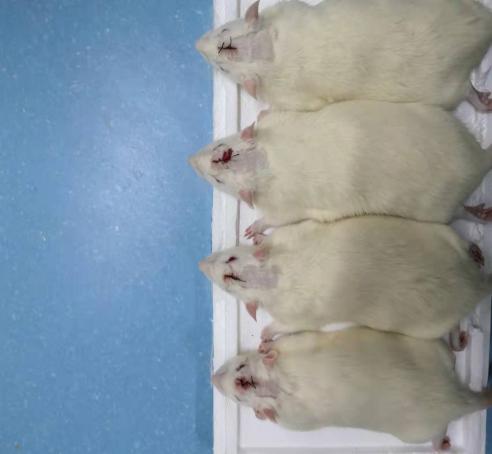


**SBI model**

Supplement: Supplementary file 2 [file DataSheet_1.docx]
